# Protein–Energy Malnutrition Is Associated with Worse Outcomes in Patients with Atrial Fibrillation: A Nationwide Analysis

**DOI:** 10.19102/icrm.2023.14082

**Published:** 2023-08-15

**Authors:** Favour Markson, Emmanuel Akuna, Iriagbonse Asemota, Gabriel Areoye, Anoz Shahi, Praise Nwachukwu, Kenneth Ong

**Affiliations:** ^1^Department of Medicine, Lincoln Medical Center, Bronx, NY, USA; ^2^Division of Cardiology, Department of Medicine, Einstein Medical Center, Philadelphia, PA, USA; ^3^Department of Medicine, John H. Stroger, Jr. Hospital of Cook County, Chicago, IL, USA; ^4^Division of Geriatrics, Department of Medicine, Vanderbilt University Medical Center, Nashville, TN, USA; ^5^Division of Cardiology, Department of Medicine, John H. Stroger, Jr. Hospital of Cook County, Chicago, IL, USA; ^6^Department of Medicine, Windsor University School of Medicine, Cayon, Saint Mary Cayon Parish, Saint Kitts and Nevis; ^7^Division of Cardiology, Department of Medicine, Lincoln Medical Center, Bronx, NY, USA

**Keywords:** Atrial fibrillation, conduction disorders, mortality, protein–energy malnutrition, underweight

## Abstract

Protein–energy malnutrition (PEM), which leads to a reduced ability of tissues to regenerate and repair themselves, may exacerbate many chronic diseases, including atrial fibrillation (AF), which occurs as a response of the heart to chronic inflammation. However, population-based studies examining the association between PEM and the prevalence and health care burden of AF are lacking. The aim of this retrospective cohort study was to estimate the impact of PEM on the prevalence and clinical outcomes of hospitalization for AF. The National Inpatient Sample (NIS) 2016 and 2017 datasets were searched for data on hospitalized adult patients with AF as a principal diagnosis; we subsequently identified AF patients with and without PEM as a secondary diagnosis using International Classification of Diseases, Tenth Revision (ICD-10), codes. The primary outcome of our study was inpatient mortality, while the secondary outcomes were hospital length of stay (LOS), total hospital cost (THC), cardiogenic shock, pacemaker insertion, successful ablation, and restoration of cardiac rhythm. Propensity score–weighted analysis was used accordingly to adjust for confounders. Out of 821,630 AF hospitalizations, 21,385 (3%) had PEM. Hospitalization for AF with PEM led to a statistically significant increase in mortality (adjusted odds ratio [aOR], 2.30; 95% confidence interval [CI], 1.93–2.75; *P* < .001) with an adjusted increase in the THC of $15,113 (95% CI, 11,246–18,980; *P* < .001), a 2-day increase in the LOS (95% CI, 1.92–2.41; *P* < .001), increased odds of cardiogenic shock (aOR, 1.36; 95% CI, 1.01–1.85; *P* = .04), and decreased odds of undergoing successful ablation (aOR, .71; 95% CI,.56–.88; *P* = .002) and achieving the restoration of cardiac rhythm (aOR, 0.56; 95% CI, 0.49–0.0.63; *P* ≤ .001) compared to those without PEM. These results indicate that PEM is associated with worse in-hospital outcomes in patients with AF. This potential association suggests that nutritional rehabilitation may be essential for improving hospitalization outcomes in AF patients.

## Introduction

Atrial fibrillation (AF) is the most frequent cardiac arrhythmia globally.^1^ In 2007, the number of newly affected individuals with AF globally was reported to be 2.3 million.^1^ However, over time, there has been a 31.6% global increase in the incidence rate, with another 3 million affected individuals documented in 2017.^1,2^ The United States alone is projected to record about 2.6 million new cases by the year 2030.^2^ The continued surge of AF and its association with medical conditions such as heart failure, stroke, chronic kidney disease, and thromboembolism, among others, predict an increased rate of morbidity and mortality in health care.

Similar to AF, studies have shown an increased prevalence of protein–energy malnutrition (PEM) among hospitalized and critically ill patients with rates of 20%–50% and 38%–78%, respectively.^3^ Despite PEM being a global health concern, it is often unrecognized and remains inadequately treated.^4^ PEM has been described as a condition of insufficient food and nutrient intake to meet the body’s requirements, leading to altered body composition (decreased fat-free mass) and body cell mass; this results in diminished physical and mental functions and impaired clinical outcomes.^5^ PEM is a condition characterized by a lack of both energy and protein and associated building blocks, such as amino acids (AAs), often resulting in damaged organs with a reduced ability to regenerate and repair themselves; therefore, it has adverse effects in many chronic diseases.

Despite the increasing reports on the impact of PEM in various acute and chronic diseases, its influence remains unrecognized in a chronic condition such as AF.

It is pertinent to investigate the potential effects of PEM on patients hospitalized for AF as this will enable interventions such as nutritional screening to reduce current high rates of morbidity and mortality in patients with AF.

This study aims to explore the role of PEM as a potential contributor to adverse outcomes in patients admitted for AF from a nationally representative sample.

## Methods

This study is reported according to the Strengthening the Reporting of Observational Studies in Epidemiology reporting guidelines.^6^

### Data source

We conducted a retrospective study of hospitalizations in 2016 and 2017 with a principal diagnosis of AF with and without a secondary diagnosis of PEM in acute-care hospitals across the United States. Hospitalizations were selected from the National Inpatient Sample (NIS) database.^7^ The NIS was created and is maintained by the Agency for Healthcare Research and Quality as the largest publicly available all-payer inpatient database in the United States. It was designed as a stratified probability sample representing all non-federal acute-care hospitals nationwide. Hospitals are stratified according to ownership/control, bed size, teaching status, urban/rural location, and geographic region. Each year, >7 million hospital stays are sampled nationwide and then weighted to estimate >35 million hospitalizations annually. Therefore, a 20% probability sample of all hospitals within each stratum is collected (excluding rehabilitation and long-term acute-care hospitals). All discharges from these hospitals are recorded and then weighted to ensure that they are nationally representative. Data from 47 statewide data organizations (46 states plus the District of Columbia) encompassing >97% of the U.S. population are included in the NIS 2016 and 2017 sampling frame. As many as 30 discharge diagnoses for each hospitalization were recorded in the NIS 2016 dataset and as many as 40 discharge diagnoses for each hospitalization were recorded in the NIS 2017 dataset using International Classification of Diseases, Tenth Revision, Clinical Modification (ICD-10-CM), codes, respectively. In the NIS, diagnoses are divided into principal and secondary. A principal diagnosis uses the main ICD-10 code for the hospitalization, while secondary diagnoses use any ICD-10 code(s) other than the principal diagnosis. As all patient data in NIS are de-identified and publicly available, the need to gain approval from the institutional review board of Lincoln Medical Center to carry out this study was waived.

### Data availability

All data used in this study are available for public access in the HCUP database (https://hcup-us.ahrq.gov/databases.jsp). ICD-10 codes used in this study are included in **Supplementary Table 1** and can be identified from the free ICD-10-CM/PCS medical coding reference website (https://www.icd10data.com/).

### Inclusion criteria and study variables

**Figure 1:**
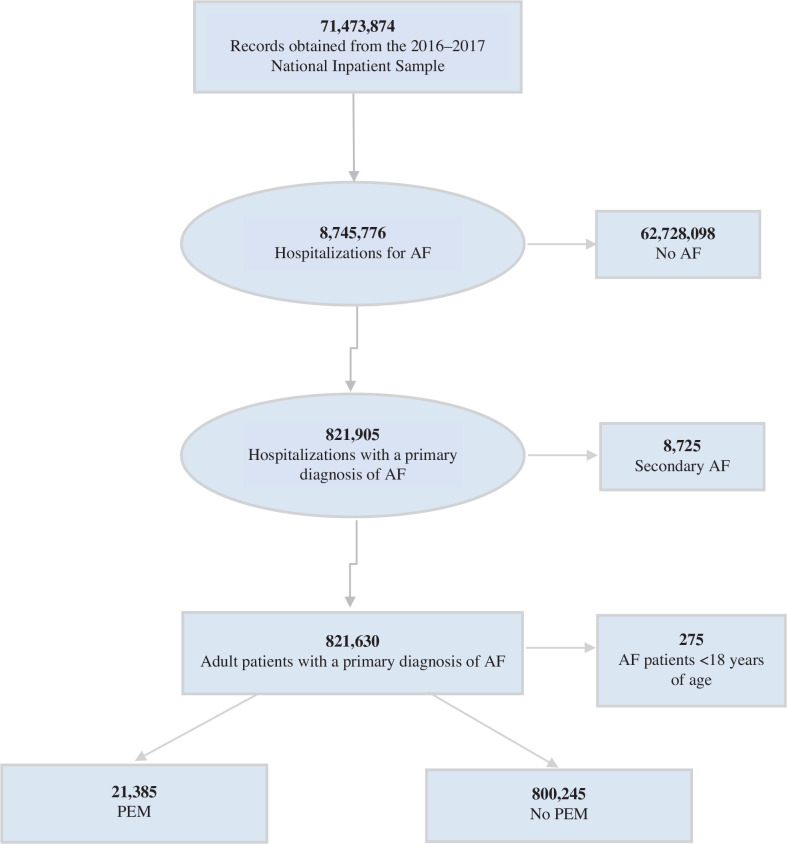
Selection flowchart. *Abbreviation:* PEM, protein–energy malnutrition.

The study population consisted of all inpatient hospitalizations recorded in the NIS 2016 and 2017 datasets for patients aged ≥18 years (selection flowchart in **Figure 1**). Study variables included age, sex, race, hospital characteristics, medical comorbidities, and primary and secondary outcomes (outlined later). We used a variety of ICD-10 codes to identify principal and secondary diagnoses (for AF, all 148.0, 148.1, 148.2, and 148.91 codes; for PEM, all E45, E46, E42, E43, E44, E44.0, and E44.1 codes) **(Supplementary Table 1)**.^8^

PEM was identified using ICD-10-CM codes **(Supplementary Table 1)**, representing cachexia, kwashiorkor, marasmus, and other types of protein–calorie malnutrition (severe, unspecified). These codes have been used in prior studies to represent PEM and have been recommended by the Academy of Nutrition and Dietetics and the American Society for Parenteral and Enteral Nutrition.^9^ However, the characteristic methodology of ascribing these codes has not been rigorously validated.

### Outcomes

The primary outcome was to compare inpatient mortality among patients principally admitted for AF with and without a secondary diagnosis of PEM. The secondary outcomes were hospital length of stay (LOS), total hospital charge (THC), pacemaker insertion, ablation, restoration of cardiac rhythm, and cardiogenic shock. The STATA software (version 16; StataCorp, College Station, TX, USA) was used for analysis. A propensity score analysis was used accordingly to adjust for confounders.

A preliminary multivariate analysis was also carried out with AF as a primary outcome and PEM as one of the many predictors from a list of covariates to determine the association between AF and PEM.

### Statistical analysis

We performed the analyses of this study using STATA version 16. We compared sociodemographic characteristics among both populations using a basic chi-squared test for categorical variables and Student’s *t* test for continuous variables.

### Propensity score analysis

Propensity score weighting is an effective methodology applied to observational studies to balance the structure of the covariates, minimizing the size of the measured and unmeasured confounders in other studies to produce an effect similar to that of randomized clinical control trials.^10^

From a list of possible covariates obtained from a prior literature search on the topic, we created propensity scores from a region of common support^3,11,12^ **(Supplementary Table 1)**.

Using inverse probability weighting, we generated propensity score weights that were incorporated into our survey sample weights as the new weights for our analysis (DuGoff et al.’s methodology).^13^ DuGoff et al.’s methodology is an analytical technique that has been used in applying propensity score models to survey data.

Following propensity score weighting, we estimated the primary and secondary outcomes by analyzing the after-treatment effect on the treated population (ATT) from our sample population.

The ATT is the difference in outcomes between the balanced groups (patients with and without PEM) following our propensity score weighting.^14,15^ The ATT was specified to accommodate the outcome variable: logistic regression was used for binary outcomes, while a generalized linear regression model was specified for continuous outcomes (THC and LOS).

*P* < .05 was considered statistically significant, and we presented effect measures (adjusted odds ratio [aOR] and mean ratio with 95% confidence intervals [CIs]) in tables and graphs using Microsoft Excel version 2205 (Microsoft Corporation, Redmond, WA, USA).

### Patient and public involvement

Patients or the public were not involved in the design, conduct, reporting, or dissemination plans of our research.

## Results

### Sociodemographic and comorbid differences

There were >71 million discharges included in the combined 2016 and 2017 NIS dataset. A total of 821,630 hospitalizations were for adult patients with a principal ICD-10 code for AF and, of these, 21,385 (2.6%) hospitalizations had PEM as a secondary diagnosis, while 800,245 (97.4%) hospitalizations did not have PEM as a secondary diagnosis. **Table 1** displays the characteristics of AF hospitalizations with and without coexisting PEM.

The PEM group was older (77.2 vs. 70.6 years, *P* < .001), with more women (58% vs. 51.3%, *P* < .001), fewer patients with diabetes mellitus (23.8% vs. 28.0%, *P* < .001) or dyslipidemia (41.3% vs. 50.5%, *P* ≤ .001), and a reduced likelihood of having hypertension (36.7% vs. 48.7%, *P* ≤ .001). However, there were more patients with chronic obstructive pulmonary disease (30.5% vs. 19.3%, *P* ≤ .001), a history of stroke (1.3% vs. 0.6%, *P* ≤ .001), hyperthyroidism (2.4% vs. 1.8%, *P* ≤ .001), malignancy (0.3% vs. 0.04%, *P* ≤ .001), peripheral vessel disease (6.7% vs. 4.1%, *P* ≤ .0001), hypothyroidism (6.7% vs. 2.9%, *P* ≤ .001), anemia (39.1% vs. 15.2%, *P* ≤ .001), and human immunodeficiency virus (0.2% vs. 0.08%, *P* ≤ .001) **(Table 2)**.

### Primary and secondary outcomes

**Figure 2:**
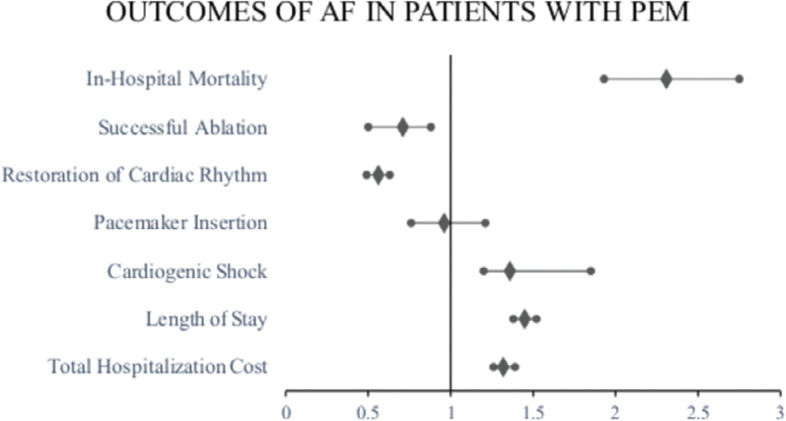
Impact of protein–energy malnutrition on in-hospital mortality, clinical outcomes, hospital length of stay, and total hospital charges in patients with atrial fibrillation. # Mean ratios; conditions adjusted for include age, sex, race or ethnicity, health insurance, health teaching status, and certain comorbidities (hyperlipidemia, hypertension, diabetes, chronic kidney disease, chronic liver disease, peripheral vascular disease, obesity, hyperthyroidism, malignancy, human immunodeficiency syndrome/acquired immunodeficiency syndrome, smoking, and prior history of myocardial infarction).

A total of 7519 adult AF hospitalization cases (0.9%) resulted in inpatient mortality: of these, 1115 (5.2%) of the deaths occurred with coexisting PEM versus 6404 (0.8%) that occurred without coexisting PEM (*P* ≤ .001). AF hospitalizations with PEM exhibited increased inpatient mortality (0.8% vs. 5.21%; aOR, 2.3; 95% CI, 1.93–2.75; *P* ≤ .001). Hospitalizations for AF with PEM had a 2.16-day increase in the adjusted mean LOS (*P* ≤ .001), higher THCs ($61,999 vs. $38,555, *P* ≤ .001), increased odds of cardiogenic shock (1.4% vs. 0.5%; aOR, 1.36; 95% CI, 1.01–1.85; *P* = .04), were less likely to have ablation (2.2% vs. 4.3%; aOR, 0.71; 95% CI, 0.5–0.88; *P* = .002), and had decreased odds of cardiac rhythm restoration (7.7% vs. 17%; aOR, 0.56; 95% CI, 0.4–0.63; *P* ≤ .001) **(Table 3, Figure 2)**.

### PEM and odds of AF

A cross-sectional study of 3,120,789 patients with PEM revealed that (21%) 648,205 patients had comorbid AF with increased odds of AF on multivariate analysis (aOR, 1.08; 95% CI, 1.07–1.09; *P* < .001); other significant comorbidities associated with an increased odds of AF include obesity (aOR, 1.63; 95% CI, 1.61–1.64; *P* < .001), chronic kidney disease (aOR, 1.30; 95% CI, 1.29–1.31; *P* < .001), hypertension (aOR, 1.40; 95% CI, 1.39–1.42; *P* < .001), hyperthyroidism (aOR, 3.64; 95% CI, 3.55–3.74; *P* < .001), chronic obstructive pulmonary disease (aOR, 1.42; 95% CI, 1.41–1.43; *P* < .001), and congestive heart failure (aOR, 4.33; 95% CI, 4.27–4.38; *P* < .001).

## Discussion

In this retrospective study, we explored the effects of PEM on hospitalized patients with AF. We demonstrated malnutrition to be associated with AF and to be a major contributing factor to these groups of patients’ financial burden, morbidity, and mortality outcomes. We showed that AF is common in the context of PEM, with approximately 21% of patients with PEM having comorbid AF. Our findings were consistent with a study done by Kang et al.,^16^ where they observed a U-shaped relationship between weight and the occurrence of AF, with both underweightness and overweightness being risk factors for the development of AF. Furthermore, in another study by Zhu et al.,^17^ the authors used nutrition assessment tools, specifically the Controlling Nutritional Status Score and the Geriatric Nutritional Risk Index, to identify malnourished patients undergoing catheter ablation for AF. They subsequently found PEM to be an independent predictor of AF recurrence after ablation.

The connection between PEM and AF has been described in relation to a reduction in myostatin, a cytokine produced by the cardiac musculature. The deficiency of myostatin in malnourished patients leads to the dysregulation of the sinoatrial node and downregulation of connexin proteins (gap-junction proteins expressed in the atrial myocardium that facilitate the effective propagation of electrical impulses). This deficiency of myostatin, therefore, enables a delayed and abnormal conduction of electrical signals in the atrium.^17,18^ The existence of this mechanism was supported by Igarashi et al.,^19^ who showed that enhancing connexin expression can cause substantial AF-suppressing activity. Similarly, Kawada^20^ demonstrated a paradoxical relationship between adiponectin levels and the incidence of AF. The author found that higher levels of adiponectin, which are prominent in malnourished patients, were independently associated with an increased risk of AF.

Our study also demonstrated the health care burden of PEM in AF, as it is associated with poorer in-hospital outcomes across almost all measured metrics. There was a significant in-hospital mortality rate, which multiple reports have corroborated. In an observational study described by Yanagisawa et al.^21^ on the predictive effects of body mass index (BMI) and AF in elderly patients, there was a significant risk of death in malnourished patients with a BMI of <18.5 kg/m^2^. Similar findings were also noted in a study by Inoue et al.,^22^ who demonstrated that, during a 2-year follow-up of 7406 patients with non-valvular AF, being underweight was an independent predictor of all-cause mortality (hazard ratio, 2.46; 95% CI, 1.62–3.69; *P* < .001).

PEM leads to a deficiency of essential nutrients that facilitates the repair of cardiac musculature in AF. One vital AA among other AAs that plays a fundamental role in cardiovascular function is arginine. The lack of this essential AA in PEM predisposes body tissues to oxidative stress and inflammatory processes associated with worsening endothelial and cardiovascular outcomes, thereby increasing morbidity and mortality.^23^ Another potential explanation of our findings could be attributed to the association between being underweight, a surrogate of frailty, and the severity of cardiac dysfunction. This theory was validated in a cross-sectional study by Carson et al.^24^ They observed cardiac cachexia as a predominant finding in advanced heart failure patients with a poor functional status and high odds of mortality.

Additionally, our study emphasized the economic burden associated with AF and comorbid PEM as patients with PEM were hospitalized for an average of four additional days and paid $22,000 more than patients without PEM. This observation might have been due to a higher occurrence of complications in this population group, which led to a prolonged hospital stay and more utilization of hospital resources. We found this to be evident in our study, where patients with AF and comorbid PEM were more likely to develop other cardiac complications, such as reduced cardiac rhythm restoration and a higher incidence of cardiogenic shock, than those without malnutrition. These complications could be due to impaired cardiomyocyte regeneration in the setting of AF and nutrient deficiencies.^11^

The worse outcomes in patients with PEM suggest malnutrition is a marker of severe disease in patients with AF and other associated illnesses such as sepsis or heart failure or a recent history of surgery.^11,25^ The knowledge obtained from this study would benefit clinicians in the stratification of patients, prognostication, and implementation of a multidisciplinary approach involving nutritional rehabilitation and dietary services to enable better treatment outcomes in patients with AF. Additionally, future studies would explore using information about acute repletion of deficient nutrients to treat hospitalized patients with AF.

### Limitations/strengths

Some notable limitations in this study were that NIS database studies are subject to all the biases associated with retrospective studies; hence, we could not ascertain the etiology of PEM in each patient. The NIS uses ICD-10 codes to characterize diagnoses and hospitalization events; hence, there was a possibility of a few coding errors. Furthermore, most of the ICD-10 billing codes used by the database failed to grade disease severity, and the study was unable to determine whether underlying disease severity affected the outcomes of patients who had AF with concomitant PEM. Another limitation of this study was the use of hospitalized patients’ data for AF rather than individual patients. Therefore, individuals hospitalized with the same principal diagnosis may have been counted multiple times. Finally, this study used an administrative database of the Healthcare Cost and Utilization Project (HCUP) that is void of valuable information such as medication usage, laboratory data, and echocardiographic findings, which limited insights into the severity of symptoms.

Despite these limitations, we used a reliable database, which has been used extensively in observational studies. We also enrolled the largest sample to date of patients with PEM and AF, which improves the power and generalizability of our study compared to other single-center studies.

## Conclusion

This study hypothesizes PEM to be a principal risk factor for worse outcomes in patients hospitalized with AF. It is essential for the collaborative efforts of health care workers and hospital leadership to implement measures that enable the prevention, early detection, and treatment of PEM to mitigate the devastating outcomes associated with PEM.

## Figures and Tables

**Table 1:** Baseline Characteristics of Atrial Fibrillation Hospitalizations According to Protein–Energy Malnutrition Status

	AF (n = 821,630)		
	Without PEM (n = 800,245)	With PEM (n = 21,385)	*P* Value
Mean age (years)	70.6	77.2	<.001
Female sex	51.3%	48.7%	<.001
Race			<.001
White	82.2%	78.8%	
Black	7.8%	10.1%	
Hispanic	6.1%	5.9%	
Asian	1.5%	2.2%	
Native American	0.35%	0.3%	
Other	2.1%	1.6%	
Charlson Comorbidity Index (points)			<.001
0	24.4%	7.9%	
1	26.4%	19.5%	
2	19.3%	21.0%	
≥3	2.1%	16.5%	
Hospital bed size			.48
Small	19.9%	20.4%	
Medium	30.5%	31.1%	
Large	49.6%	48.5%	
Hospital teaching status			.72
Non-teaching	38.0%	38.3%	
Teaching	62.0%	61.7%	
Hospital location			.32
Rural	11.2%	10.6%	
Urban	88.8%	89.4%	
Expected primary payer			<.001
Medicare	70.3%	85.3%	
Medicaid	6.2%	5.5%	
Private	21.2%	8.5%	
Self-pay	2.4%	0.6%	
Median household income (quartile)			<.001
1st (0–25th)	27.4%	30.7%	
2nd (26th–50th)	27.2%	27.6%	
3rd (51st–75th)	24.9%	22.8%	
4th (76th–100th)	20.6%	18.9%	
Hospital region			<.001
Northeast	20.3%	15.4%	
Midwest	24.1%	25.9%	
South	40.7%	38.1%	
West	14.9%	20.7%	

*Abbreviations:* AF, atrial fibrillation; PEM, protein–energy malnutrition.

**Table 2:** Comorbidities of Atrial Fibrillation Hospitalizations According to Protein–Energy Malnutrition Status

Comorbidities	Without PEM (n = 800,245) (%)	With PEM (n = 21,385) (%)	*P* Value
Dyslipidemia	50.5	41.3	<.001
Old MI	8.3	7.9	.26
Old PCI	1.1	1.1	.71
Old CABG	7.2	6.7	.23
Old pacemaker	5.5	5.1	.35
AICD	2.9	1.6	<.001
COPD	19.3	30.5	<.001
Carotid artery disease	1.3	1.6	.07
Prior stroke	0.6	1.3	<.001
Hypertension	48.7	36.7	<.001
Peripheral vessel disease	4.1	6.7	<.001
Hypothyroidism	17.3	18.7	.02
Hyperthyroidism	1.8	2.4	<.001
HIV/AIDS	0.08	0.2	<.001
Malignancy	0.04	0.3	<.001
DM type 1 or 2	28.0	23.8	<.001
CKD	17.9	26.6	<.001
Liver disease	2.9	6.7	<.001
Maintenance hemodialysis	2.1	3.8	.01
Oxygen dependence	3.4	6.3	<.001
Smoking	27.6	26.9	.33
Anemia	15.2	39.1	<.001

*Abbreviations:* AICD, automatic implantable cardioverter-defibrillator; AIDS, acquired immunodeficiency syndrome; CABG, coronary artery bypass graft; CKD, chronic kidney disease; COPD, chronic obstructive pulmonary disease; DM, diabetes mellitus; HIV, human immunodeficiency syndrome; MI, myocardial infarction; PCI, percutaneous coronary intervention; PEM, protein–energy malnutrition.

**Table 3:** Clinical Outcomes of Atrial Fibrillation Hospitalizations According to Protein–Energy Malnutrition Status

	AF with PEM (n = 17,020) (%)	AF Without PEM (n = 804,610) (%)	aOR (95% CI)		*P* Value
Primary outcome					
In-hospital mortality	5.2	0.8	2.30 (1.93–2.75)		<.001
Secondary outcomes					
Successful ablation	2.2	4.3	0.71 (0.5–0.88)		.002
Restoration of cardiac rhythm	7.7	17	0.56 (0.49–0.63)		<.001
Pacemaker insertion	1.9	1.7	0.96 (0.76–1.21)		.71
Cardiogenic shock	1.4	0.5	1.36 (1.01–1.85)		.04
			Adjusted mean difference	Adjusted mean ratio	<.001
LOS, mean, days	6.7	3.2	2.16 (1.92–2.41)	1.45 (1.38–1.52)	
Total charge, mean USD	61,999	38,555	15,113 (11,246–18,980)	1.32 (1.26–1.39)	<.001

*Abbreviations:* aOR, adjusted odds ratio; AF, atrial fibrillation; CI, confidence interval; LOS, hospital length of stay; PEM, protein–energy malnutrition; USD, United States dollars. Conditions adjusted for include age, sex, race or ethnicity, health insurance, health teaching status, and comorbidities (hyperlipidemia, hypertension, diabetes, chronic kidney disease, chronic liver disease, peripheral vascular disease, obesity, hypothyroidism, electrolyte derangement, malignancy, smoking, immunologic disorders, oxygen dependence, and prior history of myocardial infarction).

**Supplementary Table 1:** International Classification of Diseases, Tenth Revision, Codes of Principal and Secondary Diagnoses, Comorbidities, and Secondary Outcomes

Variables	ICD-10 Code(s)
Atrial fibrillation	I480, I481, I482, I4891
PEM	E45, E46, E42, E43, E44, E440, E441
Comorbidities	
Dyslipidemia	E78
Old MI	I1252
Old PCI	Z9861
Old CABG	Z951
Old pacemaker	Z950
AICD	Z95810
COPD	J41, J42, J43, J44
Carotid artery disease	I652
Prior stroke	I63
Hypertension	I10
Peripheral vessel disease	I739
Hypothyroidism	E03
Hyperthyroidism	E05
HIV	B20
Malignancy	C801
DM type 1 or 2	E10, E11
CKD	N18
Liver disease	K70, K71, K72, K73, K74, K75, K76, K77
Maintenance hemodialysis	Z992
O_2_ dependence	Z9981
Congestive heart failure	I50
Smoking	Z87891, F17200
Anemia	D50, D51, D52, D53, D55, D56, D57, D58, D59, D60, D61, D62, D63, D64
Successful ablation	0258
Periprocedural complication	I97
Restoration of cardiac rhythm	5A2204Z
Pacemaker insertion	0JH604Z, 0JH605Z, 0JH606Z
Cardiogenic shock	R570

*Abbreviations:* AICD, automatic implantable cardioverter-defibrillator; CABG, coronary artery bypass graft; CKD, chronic kidney disease; COPD, chronic obstructive pulmonary disease; DM, diabetes mellitus; ICD-10, International Classification of Diseases, Tenth Revision; MI, myocardial infarction; PCI, percutaneous coronary intervention; PEM, protein–energy malnutrition. Covariates considered in propensity score weighting/multivariate analysis: age, sex, race, primary insurance payer, hyperlipidemia, heart failure, chronic kidney disease, prior myocardial infarction, hyperthyroidism, human immunodeficiency virus, malignancy, anemia, smoking, hypertension, diabetes mellitus, and chronic liver disease.

## References

[1] Lippi G, Sanchis-Gomar F, Cervellin G (2021). Global epidemiology of atrial fibrillation: an increasing epidemic and public health challenge. Int J Stroke.

[2] Lane DA, Skjøth F, Lip GYH, Larsen TB, Kotecha D (2017). Temporal trends in incidence, prevalence, and mortality of atrial fibrillation in primary care. J Am Heart Assoc.

[3] Lew CCH, Yandell R, Fraser RJL, Chua AP, Chong MFF, Miller M (2017). Association between malnutrition and clinical outcomes in the intensive care unit: a systematic review. J Parenter Enteral Nutr.

[4] Huo Z, Chong F, Yin L, Lu Z, Liu J, Xu H (2022). Accuracy of the GLIM criteria for diagnosing malnutrition: a systematic review and meta-analysis. Clin Nutr.

[5] Cederholm T, Barazzoni R, Austin P (2017). ESPEN guidelines on definitions and terminology of clinical nutrition. Clin Nutr.

[6] von Elm E, Altman DG, Egger M (2007). The Strengthening the Reporting of Observational Studies in Epidemiology (STROBE) statement: guidelines for reporting observational studies. Lancet.

[7] NIS Database Documentation https://www.hcup-us.ahrq.gov/db/nation/nis/nisdbdocumentation.jsp.

[8] The Web’s Free 2023 ICD-10-CM/PCS Medical Coding Reference https://www.icd10data.com/.

[9] Malone A, Hamilton C (2013). The academy of nutrition and dietetics/the American Society for Parenteral and Enteral Nutrition consensus malnutrition characteristics: application in practice. Nutr Clin Pract.

[10] Kitsios GD, Dahabreh IJ, Callahan S, Paulus JK, Campagna AC, Dargin JM (2015). Can we trust observational studies using propensity scores in the critical care literature? A systematic comparison with randomized clinical trials. Crit Care Med.

[11] Charlson Comorbidity Index – An Overview | ScienceDirect Topics https://www.sciencedirect.com/topics/medicine-and-dentistry/charlson-comorbidity-index.

[12] Adejumo AC, Adejumo KL, Adegbala OM (2019). Protein-energy malnutrition and outcomes of hospitalizations for heart failure in the USA. Am J Cardiol.

[13] DuGoff EH, Schuler M, Stuart EA (2014). Generalizing observational study results: applying propensity score methods to complex surveys. Health Serv Res.

[14] Becker SO, Ichino A (2002). Estimation of average treatment effects based on propensity scores. Stata J.

[15] Abdia Y, Kulasekera KB, Datta S, Boakye M, Kong M (2017). Propensity scores based methods for estimating average treatment effect and average treatment effect among treated: a comparative study. Biom J.

[16] Kang SH, Choi EK, Han KD (2016). Underweight is a risk factor for atrial fibrillation: a nationwide population-based study. Int J Cardiol.

[17] Zhu S, Zhao H, Zheng M, Peng J (2021). The impact of malnutrition on atrial fibrillation recurrence post ablation. Nutr Metab Cardiovasc Dis.

[18] Lübkemeier I, Andrié R, Lickfett L (2013). The Connexin40A96S mutation from a patient with atrial fibrillation causes decreased atrial conduction velocities and sustained episodes of induced atrial fibrillation in mice. J Mol Cell Cardiol.

[19] Igarashi T, Finet JE, Takeuchi A (2012). Connexin gene transfer preserves conduction velocity and prevents atrial fibrillation. Circulation.

[20] Kawada T (2016). Total and high-molecular-weight adiponectin are associated with incident atrial fibrillation. Heart.

[21] Yanagisawa S, Inden Y, Yoshida N (2016). Body mass index is associated with prognosis in Japanese elderly patients with atrial fibrillation: an observational study from the outpatient clinic. Heart Vessels.

[22] Inoue H, Kodani E, Atarashi H (2016). Impact of body mass index on the prognosis of Japanese patients with non-valvular atrial fibrillation. Am J Cardiol.

[23] Goni L, Razquin C, Toledo E (2022). Arginine catabolism metabolites and atrial fibrillation or heart failure risk: 2 case-control studies within the Prevención con Dieta Mediterránea (PREDIMED) trial. Am J Clin Nutr.

[24] Carson MA, Reid J, Hill L (2022). Exploring the prevalence, impact and experience of cardiac cachexia in patients with advanced heart failure and their caregivers: a sequential phased study. Palliat Med.

[25] Williams DGA, Molinger J, Wischmeyer PE (2019). The malnourished surgery patient: a silent epidemic in perioperative outcomes?. Curr Opin Anaesthesiol.

